# A Systematic Bibliometric Review Analysis of Research on the Use of Waste Rubber Tyres in Building and Construction Materials and Their Applications

**DOI:** 10.3390/polym17182480

**Published:** 2025-09-13

**Authors:** Rosnery Castillo, Aleix Alva, Oriol París-Viviana, Montserrat Bosch

**Affiliations:** 1 Department of Architectural Technology, Barcelona School of Building Construction, Universitat Politècnica de Catalunya, Av. Doctor Marañon 44, 08028 Barcelona, Spain; oriol.paris-viviana@upc.edu (O.P.-V.); montserrat.bosch@upc.edu (M.B.); 2 Departamento de Técnica, Facultad de Arquitectura y Diseño, Universidad de Panamá, Campus Dr. Octavio Méndez Pereira, Av. Transístmica, Ciudad de Panamá P.O. Box 3366, Panama; 3 Department of Architectural Technology, ETSAB—Barcelona School of Architecture, Universitat Politècnica de Catalunya, Av. Diagonal, 649, Les Corts, 08028 Barcelona, Spain; aleix.alva@upc.edu

**Keywords:** bibliometric analysis, building materials, construction materials, waste rubber tyre (WRT), end-of-life tire or tyre (ELT), crumb rubber tyre (CRT), concrete

## Abstract

This systematic bibliometric review, conducted in accordance with the PRISMA methodology, examines the literature on waste rubber tyres (WRTs) and their applications, measuring correlations between several standard metrics and identifying gaps that may stimulate new research. Using a thirty-year database from Scopus, both numerical and graphical results highlight key aspects, including geographic distribution, journal analysis, keywords, and three main research categories: environment, application, and property. Publications have grown exponentially at a rate of 17% per year. Research is concentrated in India and China, with Africa and Central America lagging behind. The United States leads in impact, while publication volume correlates moderately with gross domestic product (GDP), but negatively with international collaboration. Notably, several leading countries in automobile and tyre production have a limited academic presence in WRT research. The most prominent studies focus on material development; however, there remains a lack of practical applications for these materials. Recent trends show growing interest in WRTs within engineering, materials science, and environmental science, with multidisciplinary approaches emerging. A keyword analysis indicates a steady evolution in end-of-life tyres (ELT) research over recent years. In environmental studies, interest in ELT recycling methods has grown, though aspects such as toxicity, life cycle analysis, and leaching remain relatively unexplored. In terms of applications, research is primarily focused on pavements, while areas such as facades, blocks, and roofs remain under-studied. Finally, in material property studies, most research addresses compressive strength, while critical areas such as fire resistance, impact resistance, and thermal testing offer promising avenues for future research.

## 1. Introduction

According to the Sixth Assessment Report (AR6) of the United Nations (UN) Intergovernmental Panel on Climate Change (IPCC) [1], the building sector was responsible for 21% of the global greenhouse gas (GHG) emissions in 2019. From this percentage, 18%, meaning 3.78% globally, was attributed to the carbon dioxide (CO_2_) emissions generated on-site during the production of cement and steel intended for the construction and/or refurbishment of buildings [2]. However, according to the report “Building Materials and The Climate: Constructing a New Future” the built environment sector was contributing 37% of energy-related carbon emissions by 2022 [3].

Demographic projections predict a continuous growth over the next few decades, which would result in a significant demand for new constructions and infrastructure to meet the needs of an expanding population [4]. Although concrete is renowned as one of the primary materials in the construction industry, its production requires high energy consumption [5]. Moreover, approximately 6 billion tonnes of natural coarse aggregate (NCA) and natural fine aggregate (NFA) are utilised annually [6]. This extensive reliance on natural aggregates presents critical sustainability and conservation challenges for natural resources, thereby becoming one of the primary threats to the environment [7]. According to the UN, the global demand for sand and gravel stands at around 50 billion tonnes per year, making them the second most utilised resources in the world after water [8].

According to the World Bank Report “What a Waste 2.0: A Global Snapshot of Solid Waste Management to 2050” in 2016 [9], 5% of global emissions originate from the management of solid waste, excluding transportation. The report warns that, unless immediate action is taken in the management of solid waste, it will increase by 70%, rising from the 2010 million tonnes recorded in 2016 to 3400 million tonnes.

One of the global concerns in terms of solid waste is the management of end-of-life tyres (ELT), a non-biodegradable material [7]. In 2018, global tyre production was estimated at 17.1 million tonnes [10]; however, the Global ELT Management Report [11] in 2019, indicated 1.7 million tonnes of ELT were recycled across categories such as civil engineering and backfilling, energy recovery, material recovery, and others. The search to incorporate waste into construction materials has facilitated the integration of ELT into the building sector [12,13].

Currently, there has been a significant increase in the use of WRT as an alternative aggregate [14,15,16,17]. Although several existing reviews in the literature already offer focused perspectives on the comparative substitution of natural coarse aggregates (NCA) or natural fine aggregates (NFA) with recycled materials, such as crumb rubber from tyres (CRT) [18,19,20,21], the recycling methods for ELTs and the mechanical properties of these upcycled materials [17,19,20] lack a detailed bibliometric analysis of all these data [22,23,24].

In alignment with other bibliometric studies in the building construction field addressing (1) bio-based building materials [25]; (2) construction and building technology [26]; (3) “asphalt pavement and bitumen materials” [27]; (4) “waste for production of bioproducts” [28]; (5) “polymers” [29,30,31], this study aims to systematically analyse the published literature on construction materials derived from end-of-life tyres (ELTs) to identify their applications, the methods employed, and the main research objectives in this field, ultimately seeking to understand what is being investigated, how, and for what purpose ELTs are used in construction, as well as the findings obtained.

This is achieved through a comprehensive review of the academic literature, mapping the primary research trends, the emerging countries, the most relevant authors, and the most cited publications. This study can provide countries with a basis from which to evaluate the effectiveness of their national policies regarding tyre waste, by comparing the outcomes of their research programmes with those of other nations. Furthermore, it can serve to enable the scientific community to identify potential synergies between institutions, generate more scientific collaboration networks, and provide a general perspective that can help identify new research lines.

## 2. Methodology

**Figure 1:**
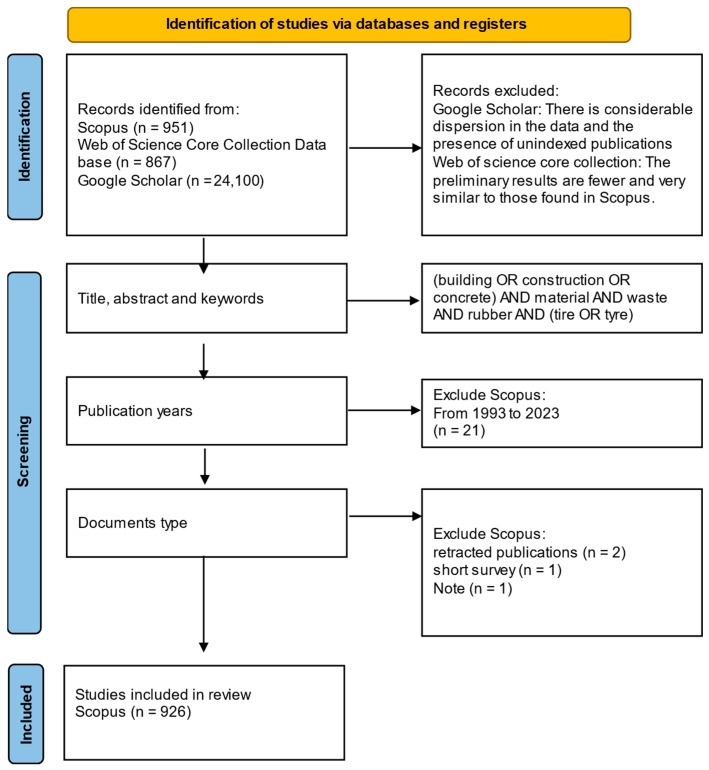
A flow diagram of the bibliometric analysis is presented.

There are different approaches to bibliometric analysis, each with its own set of metrics [32,33,34,35]. This study focuses on the formulation of four key interrogatives: (1) what (keywords and emerging trends); (2) when (the evolution of indexed documents across a period of time); (3) where (the geographical distribution of the published literature and the collaborations between countries); (4) and who (journals, institutions, and authors through publishing and collaboration networks) [25,36]. As shown in Figure 1, this systematic review was carried out in accordance with the Preferred Reporting Items for Systematic Reviews and Meta-Analyses (PRISMA) guidelines [31,37]. This study was preregistered on Preprints.org with the identifier preprints-174779.

### 2.1. Data Bases and Keywords

A set of keywords was selected in order to establish a precise search [25]. In order to curate this set, given that the main focus is on the use of ELT, particular emphasis was given to the processing of this waste material, but also to the identification of studies that report its output to the construction and building industry in the form of a product. Accordingly, “rubber” and “material” were direct choices for the subject of study. In order to delimitate the field of study, “construction”, “building” and “concrete” were included with an OR logical operator. Given the abundance of studies of rubber in this field, more ELT specificity was targeted by adding the term “waste”. Additionally, to avoid partial results due to the orthographic variation in English, both “tyre” and “tire”, from British and American English, respectively, were merged with a union. The final search string, including logical operators, is shown in Table 1.

The search was carried out on 3rd July 2024, and results were filtered by date, selecting results from the interval [1993, 2023] and only considering whole years. For that process, three bibliographic databases were used: Google Scholar, Web of Science (WoS) and Scopus. Results from Google Scholar were discarded due to the presence of unindexed publications.

For the search in WoS, the “Web of Science Core Collection” tool was used, due to its wide variety of advanced search options (keywords, cited reference count, funding information, affiliation, etc.,) and its access to the complete WoS databases. The search was performed on the “topic” field, which includes title, abstract, keyword plus (Keyword plus are words or phrases that frequently appear in the titles of an article’s references, but do not appear in the title of the article itself) and author, and 862 publications were obtained. The search in Scopus was carried out in the “Article title, Abstract, Keywords” field, obtaining 930 publications. These two sets exhibited a high degree of similarity, and the latter was chosen due to its slightly greater volume. Two retracted publications, a short survey and a note were removed from the final set, leaving a total of 926 documents.

### 2.2. Data Compilation, Processing and Analysis

Data from Scopus were exported in Comma Separated Value (CSV) format and were processed and visualised with Microsoft Excel; VOSviewer (Version 1.6.20), developed by the Centre for Science and Technology Studies (CWTS); and Biblioshiny by Bibliometrix (version 4.1.2) [38,39] with Rstudio (4.4.0) [40].

Various metrics were employed to assess each of the outcomes: (1) H index by Jorge Hirsch, which seeks to balance the total number of citations received in relation to scientific output [41]; (2) average citations per document, referring to the period 1993–2023; (3) evolution of number of publications per year; (4) publications by type and languages; (5) scientific production by country and city; (6) collaborations by country; (7) research activity by institution; (8) authors with highest production rate; (9) most cited papers; (10) journal production rate; (11) research areas; (12) temporal evolution of keywords; and (13) areas of study. The complete bibliometric dataset and bibliography used for this study can be found in the CORA Research Data Repository [42].

### 2.3. Correlation Analysis

For some pairs of metrics, two correlation coefficients were considered. On the one hand, Pearson’s correlation coefficient, in order to quantify the degree of alignment between each pair, while taking into account the magnitude of each metric. On the other hand, Kendall’s correlation coefficient, particularly τB (which accounts for ties), was also provided in order to quantify the alignment between the ranks of the metrics. While both coefficients broadly agree, some nuances can be drawn from their distinction. One of the reasons is that journal ranks play a very strong role in how academic assessment is carried out. These correlation coefficients were calculated with the R programming language, after processing the data with LibreOffice’s Calc software (https://www.libreoffice.org/discover/calc/, accessed on 30 August 2024). For both coefficients, values of 1, 0 and −1 indicate perfect alignment, inverse alignment, and independency, respectively.

Non-linear fits were carried out with Grace software (https://plasma-gate.weizmann.ac.il/Grace/, accessed on 30 August 2024). The exponential function is written as y = a_0_·exp(a_1_(x − a_2_)), and the cumulative generalised Pareto distribution as y = 1 − (1 + a_0_(x − a_1_)/a_2_)^(−1/a_0_)^. In both cases, the parameters to be fitted are written in aj form.

## 3. Results and Discussion

### 3.1. Temporal Evolution of the Number of Publications andPublications by Type and Languages

The initial publications on WRTs date back to 1973 [43], 1974 [44], and 1975 [45]. In the first of them, the reutilisation of different materials, including tyres, was discussed; in the second, methods of converting tyre waste into fuel were explored; in the latter, possible future applications in the construction and civil engineering industries were proposed, although the first patent on rubber crumb reinforced cement concrete, filed by Frankowski, was still 17 years ahead [46].

**Figure 2:**
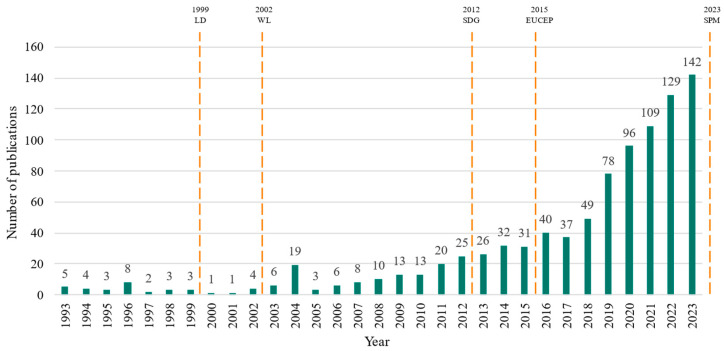
Evolution of the number of publications per year according to Scopus data, with a dashed line representing some milestones: 1999 Landfill Directive (LD), 2002 Waste at Landfills (WL), 2012 Sustainable Development Goals (SDG, 2015 European Union’s Circular Economy Package (EUCEP), 2023 Synthetic Polymer Microparticles (SPM).

Figure 2 shows the time evolution of the number of publications by year. The graph shows a markedly exponential growth. A non-linear fit was performed against the expression N = a_0_·exp(a_1_(t − a_2_)) with the resulting values a_0_= 0.70, a_1_ = 0.17 and a_2_ = 1992.37 years and with the statistical values of chi-square = 915.179, correlation coefficient = 0.990389 and RMS relative error = 0.627477. The parameter a_1_, which indicates a growth rate of 17% per year, predicts about 500 publications per year by 2030.

Significant global events before and during this period that could have spurred interest and research in the area include: the Brundtland report (1987) [47]; the UN Conference on Environment and Development (Earth Summit), held in Río de Janeiro in 1992 [48]; the emergence of the UN SDGs (Sustainable Development Goals) in 2012 [49]; and the European Union’s Circular Economy Package, a regulatory framework where ambitious recycling targets and promoted research into innovative recycling processes, including tyre recycling, was adopted in December 2015 [50].

A significant event is worth highlighting. In December 2002, the European Union established criteria and procedures for the acceptance of waste at landfills [51] for its 1999 Landfill Directive [52], which regulated that landfill facilities would not accept used tyres or waste which were liquid, flammable, explosive or corrosive. This regulation likely was the reason behind the surge of publications about WRT in 2004 shown in Figure 2, as industries and researchers focused on finding alternative ways to manage and recycle tyres. In September 2023, Commission Regulation (EU) 2023/2055, regarding synthetic polymer microparticles, introduced restrictions on the manufacture and marketing of microplastics [53]. This regulation addresses the toxicity and leaching potential of these particles, highlighting the negative impacts they may have on both ecosystems and environmental health.

Driven by these global initiatives, countries have implemented regulations to promote sustainable development. In the case of ELTs, laws such as the Extended Producer Responsibility (EPR) have been standardised in some countries, aiming to recycle at least 90% of tyres by 2030 [54]. Additionally, there are regulations focused on tyre management, restrictions on tyre disposal in landfills, promoting tyres as a raw material, and preventing the importation of ELTs. Various measures for ELT management have been implemented across different regions of the world [11]. Some examples are the European Union, where several directives and standards have been established [52,55,56,57]; in China, specific provisions for ELT management are also in place [58,59,60], in the United States, legislation includes guidelines for tyre management and recycling [61,62], and in Brazil, CONAMA Resolution 416 of 2009 regulates the treatment of these materials [63].

**Figure 3:**
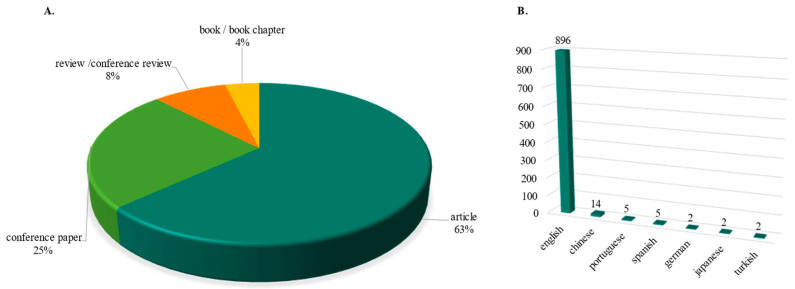
Publications by type and language. (**A**) Pie chart with the distribution of publication types. (**B**) Distribution of languages.

The obtained 926 publications originate from 347 different sources, comprising 66% articles, 25% conference papers, 8% review and conference reviews and 4% book or book chapters. As illustrated in Figure 3, 96% of the publications are in English, 1.5% are in Chinese and the remaining 2.5% are in Portuguese, Spanish, German, Japanese, and Turkish.

### 3.2. Geographical Analysis

**Figure 4:**
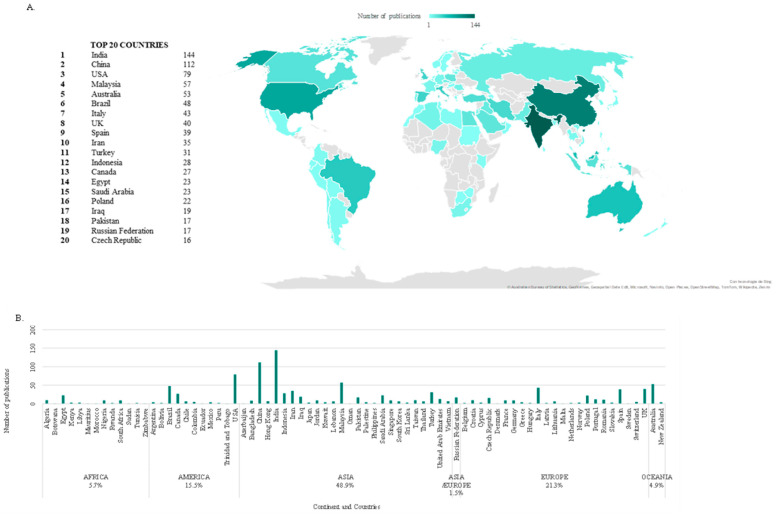
Global scientific output by country: national and continent-specific contributions to research publications. (**A**) Heat map of countries with publications and list of the 20 countries with the most publications. (**B**) Scientific output categorised by continent and the 76 countries that have contributed to the item.

Figure 4A,B show the world distribution of the selected publications and the top 20 list, respectively. The results show that only 30% of the 76 contributing countries have 11 or more publications. The first country in the list, India, just 0.5% of all nations, already represents more than the 15% of the publishing output. Following this Pareto-based statistic, it can be seen how 3%, 5% and 7% of the countries represent 50%, 70% and 80% of the academic production, respectively. Fitting these data to the cumulative distribution function of the generalised Pareto distribution, values of the shape and scale parameters of 0.33 and 3.39 are obtained, respectively. The former, an indicator of inequality, reveals that this metric is heavily tailed, i.e., inequitably distributed.

The alignment of this list with the gross domestic product (GDP) [64] ranking is substantial but not complete. On the one hand, several high-GDP-ranked countries like Germany (3rd), Japan (4th) or France (7th), do not appear among the 20 countries with the most publications on the subject. On top of that, the first two are top car producers [65], and the latter is the world’s leading tyre producer offshore [66].

On the other hand, there are significant examples of relatively low-GDP countries being included in the list, like Malaysia (36th), Egypt (42nd), Iran (34th), the Czech Republic (45th) or Pakistan (43rd). India, being ranked as 5th in GDP, appears as 1st in the publishing list. A Pearson correlation analysis between GDP (International Monetary Fund 2024 forecast) and the list from Figure 4 gives a 0.55 coefficient, indicating a moderate correlation between these two metrics.

However, it is worth mentioning that, according to the European Tyre & Rubber Manufacturers Association (ETRMA) [67], the end-of-life tyre management system in Germany is classified as a “Liberal System” (free market). This could be one of the reasons why Germany does not appear among the countries with the highest number of publications. Although Japan and France are not among the leading countries in publications on this topic, they have also contributed research in this area.

Figure 4 shows the top 20 countries with the highest number of scientific contributions over the last 30 years. The most productive continent has been Asia, accounting for 48.9% of publications, followed by Europe with 21.3%, the Americas with 15.5%, Africa with 5.7% and Oceania with 4.9% of publications.

**Figure 5:**
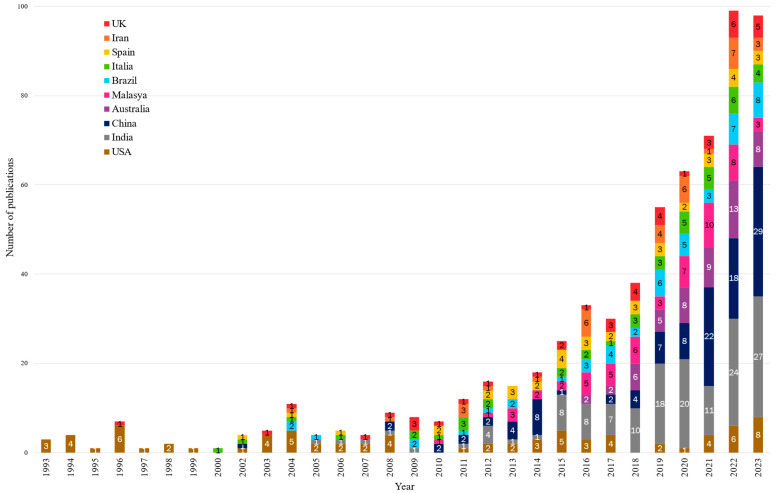
Temporal trends in research output: evolution of publication patterns in the top 10 productive countries. Arranged vertically by country colour.

Figure 5 shows a breakdown of the evolution of the total number of publications by top-10 publishing country. The data reveal that the exponential growth is mainly due to contributions by China and India. The rest of countries present alternating or stationary trends. In particular, contributions from the USA, the most prominent country in the data until 2008, revealing a premature commitment in ELT reutilisation, have not significantly grown since then. Malaysia and Australia average between 1 and 13 publications per annum, whereas Brazil, Italy, the UK, Spain, and Iran typically range from 1 to 8 yearly publications.

While the number of publications can reveal a country’s scientific production, it is necessary to consider other metrics to evaluate the impact of research. Next, the analysis of the following metrics is presented: total publications, total citations received, average citations per document, local H-Index (the country’s H-Index in this search) and the percentage of international co-authorship (the proportion of total publications that have collaborated with other countries).

Table 2 shows the countries with the highest citations, with the USA holding the top position with 5395 citations and an average of 68.3 citations per document over the past 30 years. This value is accompanied by a local H-Index of 29, indicating that USA is the country with the most significant impact in the field. Despite having 45% fewer publications than India, articles from the USA appear to have greater impact. India, with the largest number of publications, has 2896 citations, although its local H-Index is 24 and it trails the USA by 46% in terms of citations received.

Both Pearson’s and Kendall’s τ coefficient evaluation are performed on metrics from Table 2 to assess their correlation. The resulting matrices are shown in Table 3. Most metrics are weakly to moderately correlated, except “local H-index” and “Number of citations”, which show a very significant (0.86 | 0.75) alignment (correlation coefficients are given with the format Pearson | Kendall). Remarkably, the total number of publications and the total number of citations only show moderate alignment (0.42 | 0.33). Another interesting conclusion is that correlations between the number of citations and the rest of metrics are systematically lower when measured by rank.

The total number of publications negatively and moderately (−0.37 | −0.24) correlates with the average citations per document. This suggests a preliminary hypothesis that a country’s impact is not necessarily tied to a high volume of articles, but rather to the quality and relevance of its publications. It is worth mentioning that there is no consensus on how quality can be measured. Indicators measuring reputation and popularity are usually taken as proxies for it, but this identification can hide a pernicious inversion. High-reputation and/or high-resource countries could monopolise the information channels in such a way that, independently of quality, most of the quantitative metrics are accumulated for their benefit. With that mechanism, an advantageous position can lead to more popularity, and in that case, taking it as a proxy for quality represents a harmful bias.

**Figure 6:**
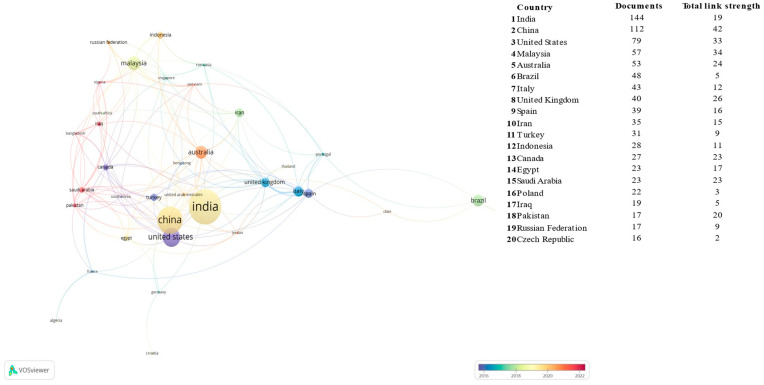
Diagram showing co-authorship relationships in publications based on the authors’ countries of affiliation, with clusters distinguished by colour and the main connections between them. The diameter of each circle represents the number of publications, and the total strength indicates the overall number of links established with other countries. Created using VOSviewer software.

Figure 6 shows the collaboration network by countries. India, the country with most publications, has the smallest collaboration percentage (16%). This is the main factor behind the negative correlation between co-authorship and number of publications (−0.52 | −0.38). This result can be understood by considering the following points. Firstly, India is a country with a strong emphasis on education, which plays a pivotal role in enhancing social mobility. Secondly, with the exception of a few top institutions, most universities lack support systems to foster internationalisation [68]. Thirdly, there is a significant motivation among Indian academics to relocate abroad, leading to publications with foreign affiliations. Lastly, a prevalent bias, which views this country’s research as having lower quality, can result in fewer international connections with other prejudiced countries [69]. This point may explain why, in the case of China (a country not subject to this prejudice), this negative correlation is not observed.

### 3.3. Research Activity of Institutions

Geographic data are based on affiliations declared by authors. The number of registered affiliations is 744, of which 697 have less than 10 articles on this topic. In other words, 93% of affiliations have fewer than 10 published articles, illustrating the Pareto principle, where the majority of publications are produced by a minority of affiliations.

The top 10 publishing affiliations were selected for further analysis. Table 4 shows data for four metrics: total number of publications, total number of citations received, the local H-index and the average citations received per document. For each top publishing affiliation, four metrics are given, plus its country of origin: (1) the total number of publications; (2) the total number of citations received by the affiliation; (3) the local H-index; (4) and the average numbers of citations per document, which is the ratio between the second and the first metrics.

Applying again a Pearson’s | Kendall’s evaluation, it can be seen that, regardless of the magnitude of the coefficient, all metrics are positively correlated. The highest values of this parameter are obtained between the number of citations and the average citations per document (0.99 | 0.91) and between the total number of publications and the local H-index (0.83 | 0.69). The total number of publications and the average number of citations are significantly correlated (0.73 | 0.30), though Kendall’s parameter reveals that, in terms of rank, a high rank in the number of citations does not necessarily lead to a high rank in the average citations per document.

The most contributing institution is MNIT, with 18 publications, and it is located in the most contributing country, India. The second most contributing institution is RMIT University, with 17 publications, which amounts to 32% of all documents from Australia. MNIT has three publications with more than 150 citations, “Strength, Abrasion and Permeation Characteristics of Cement Concrete Containing Discarded Rubber Fine Aggregates”, “Performance of High Strength Rubberized Concrete in Aggressive Environment” [70,71] and “Recycling of Dimensional Stone Waste in Concrete: A Review” [72]. Significantly, two of the three are co-authored with the UK. RMIT University has 17 publications and collaborates with China, Egypt, India, Thailand and Vietnam. Its most cited article, “Recycling Waste Rubber Tyres in Construction Materials and Associated Environmental Considerations: A Review” [73], is co-authored with Thailand and has a total of 351 citations.

Malaysia is represented by UTHM with 15 publications and UTM with 13 publications, together contributing 49% of Malaysia’s total output. UTHM collaborates internationally with Canada, Indonesia, Russia, Norway, Pakistan and Singapore. Its most cited publication is “Properties of Cement Mortar Containing Rubber Ash as Sand Replacement” [74], with 30 citations. UTM, with collaborations in Russia, Canada, Norway, China, Ecuador, Iran, Pakistan, Singapore, the United Arab Emirates and Vietnam, has a notable publication with more than 100 citations “Application of Polymer, Silica-Fume and Crushed Rubber in the Production of Pervious Concrete” [75].

In China, SEU and the Ministry of Education of the People’s Republic of China account for 28% of the country’s publications. SEU collaborates with Australia and the USA, and its most cited paper is “Evaluation of Optimum Mixing Conditions for Rubberized Asphalt Mixture Containing Reclaimed Asphalt Pavement” [76], with 61 citations. Meanwhile, the Ministry of Education of the People’s Republic of China’s most cited publication, in collaboration with Curtin University, Australia, is “Fracture Behavior of a Sustainable Material: Recycled Concrete with Waste Crumb Rubber Subjected to Elevated Temperatures” [77], with 99 citations.

Islamic Azad University of Iran, Sapienza University of Rome in Italy, and UNICAMP in Brazil are tied with approximately 12 publications each, representing 34%, 28%, and 25% of their countries’ output, respectively. Islamic Azad University collaborates with Australia, New Zealand, Greece, Vietnam, Portugal, Malaysia, China, Dubai and Ecuador. Its most cited paper, “Application of Polymer, Silica-Fume and Crushed Rubber in the Production of Pervious Concrete” [75], has 147 citations, co-authored with Central South University, China. Sapienza University of Rome collaborates with the United Kingdom, Jordan and Belgium. Its most cited publications are “Extrusion-Based Additive Manufacturing of Concrete Products: Revolutionizing and Remodeling the Construction Industry” [78] with 66 citations, and “Reducing the Emission of Climate-Altering Substances in Cementitious Materials: A Comparison between Alkali-Activated Materials and Portland Cement-Based Composites Incorporating Recycled Tire Rubber” [79], with 65 citations. UNICAMP has no international collaborations, and its most cited article is “Effects of Spheroid and Fiber-like Waste-Tire Rubbers on Interrelation of Strength-to-Porosity in Rubberized Cement and Mortars” [80] with 56 citations. Finally, CTU in the Czech Republic has 11 publications and collaborations with Poland. Its most cited article is “Eco-Friendly Concrete with Scrap-Tyre-Rubber-Based Aggregate—Properties and Thermal Stability” [81], with 85 citations.

### 3.4. Author Evolution and Documents

A total number of 2680 authors have been found in this literature, where 83% has published only once and only 35 authors have contributed to 5 or more publications. Table 5 shows a list of the top 10 most published authors. To further characterise this list, the global Scopus H-index, the total number of publications and the total number of citations are included as additional metrics. These last three metrics include publications outside the selected list of this study, allowing us to analyse how publications in this study’s field correlate with broader academic scopes.

For these authors, there are 25.2 citations per article derived from the selected list, and 16.9 citations per article derived from the global numbers. This could indicate that, in broad terms, the academic profit in this study’s field would be above the average of all considered topics. However, a closer inspection reveals that the ratio between the former and the latter metrics is 1.32 ± 0.52, so the difference between these two quantities is not very significant. What can be concluded is that the utilisation of waste tyre is a field with significant engagement, above or at least of the same order as the rest.

**Figure 7:**
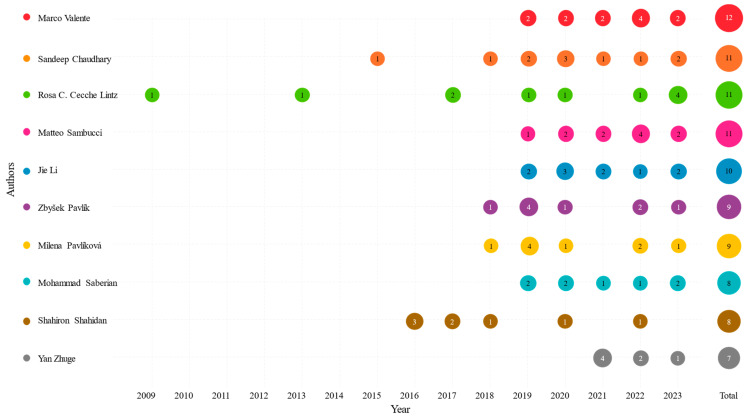
Shows the temporal evolution of the top 10 most published authors.

As demonstrated in Figure 7, the temporal progression of the top 10 most prolific authors indicates a substantial augmentation in academic productivity commencing from 2019.

### 3.5. Global vs. Local Citations

Global citations are defined here as the absolute number of citations that an article from the selected list has received, whereas local citations are defined as the number of citations coming from the very same list. This distinction, which can be computed with Biblioshiny’s algorithm allows to compare the global vs. the local impact of each publication. Table 6 shows the 10 most globally cited publications with their corresponding local metric. Pearson’s correlation coefficient between these two metrics is 0.95, indicating that they are strongly correlated, and that local citations are a good proxy for their global counterparts.

In the description (Table 6) of the most cited articles we have, Siddique et al. [82] detail various methods for recycling tyres and describes the properties of both fresh and hardened concrete, also highlighting the benefits of using magnesium oxychloride cement as a binder for rubberised concrete mixes. Zaher and Fouad [83] present an experimental campaign developing a characteristic function to quantify the reduction in the strengths of crumb rubber concrete (CRC) and suggest its use for non-structural purposes, such as lightweight concrete walls, building facades, and architectural units. Khaloo et al. [84] performed an experimental study in which natural aggregates were replaced with crumb rubber tyres (CRT), and tests were carried out on strength and acoustic absorption. Aiello et al. [85] examine the replacement of natural aggregates with CRT and analyse the post-cracking behaviour of rubberised concrete, showing energy absorption and ductility indices comparable to fibre-reinforced concrete. Onuaguluchi et al. [86] describe the substitution of varying percentages of natural aggregate with pre-treated CRT and the addition of silica fume as a cement substitute. The study includes tests on concrete strength, permeability, and durability. Issa et al. [87] discuss how CRT reduces concrete strength and the cost of processing this waste, in addition to conducting an experimental campaign substituting aggregates with CRT. The work of Fattuhi and Clark [88] involves preparing mortar and concrete mixes using various proportions of CRT or low-grade rubber and conducting tests on strength and physical properties. Reda Taha et al. [89] examine strength and fracture tests on CRC in an experimental campaign. Thomas et al. [70] include CRT tests to determine the compressive strength, flexural strength, abrasion resistance, microstructure, water permeability, and sorption in concrete samples. Batayneh et al. [90] focus on substituting fine aggregate with CRT and recommend that authorities promote the use of waste in construction, and that the industry should seek ways to incorporate this waste.

Among the ten most cited publications, there is a clear trend towards the development of experimental articles with a strong focus on their mechanical properties. However, only one of them promotes the use of concrete with rubber in the construction industry and provides recommendations to authorities on regulations, highlighting a notable deficiency in the application of construction elements made from this new material. This reflects the need not only to focus on the material’s mechanical properties but also on how these materials can be applied within the industry.

### 3.6. Journal Analysis

This section provides an analysis of the 347 journals involved in the selected database, of which 90% feature less than five publications. The study focuses on the top 10 most contributing journals, and extracts these metrics for each of them: (1) number of published documents; (2) total number of received citations; (3) average citations per document; (4) local H-index of the journal; (5) global H-Index of the journal; (6) Cite Score 2023 (average number of citations over a three-year period); (7) Scimago Journal and Country Rank (SJR); (8) Source Normalised Impact per Paper (SNIP) (number of citations relative to the average number of citations in its field.); and (9) Quartile Score (relevance of the journal against other journals in the same area).

Table 7 shows these journals and their corresponding metrics, these 10 journals represent only 3% of the total journals addressing this topic but contribute 34% of the publications.

Table 8 shows the Pearson’s and Kendall’s coefficient matrices for these metrics. The data show a very strong correlation between Cite score 2023, SJR 2023 and SNIP 2023. The global and local H-indices have a fairly strong correlation (0.81 | 0.67) and they are both reasonably correlated with the three aforementioned metrics. Remarkably, the number of papers and their average citation number seem to be completely independent. In other words, the publication volume of a journal does not correlate with its impact. Regarding differences between correlations based on magnitude (Pearson) and rank (Kendall), both H-indices prominently reduce their correlation with the number of papers when analysed by rank.

In Table 7, the leading journal in terms of number of publications is “Construction and Building Materials”, with a total of 6449 citations; it also ranks highest in terms of local H-Index (47). The second-ranked journal, Journal of Cleaner Production, has a local H-index of 28, though it is not the second most cited. It is important to note that a higher number of citations does not necessarily equate to greater impact. Even so, as seen in Table 8, there is a strong correlation between the number of citations and the local H-index (0.94 | 0.94). For this reason, as shown by various metrics in the table, the Journal of Cleaner Production demonstrates a greater impact than Waste Management, according to the local H-index.

The third and fourth positions are held by “Lecture Notes in Civil Engineering” and “IOP Conference Series: Materials Science and Engineering”, with 35 and 28 publications, respectively. These journals, which publish conference papers, conference reviews, and book chapters, have local H-indices below 10. For “IOP Conference Series: Materials Science and Engineering”, global data are from 2021, as Scopus implements rigorous quality control procedures and journals not meeting these standards are removed from the database; hence, the journal lacks a Quartile Score, and its global metrics are from 2021.

In the fifth and sixth positions are “Materials” and “Materials Today: Proceedings”, respectively. Although both journals have 26 and 22 publications, “Materials” has 612 citations, which is over three times the citations of “Materials Today: Proceedings”, and an average number of citations per document of 23.5, whereas “Materials Today: Proceedings” has 158 citations and an average of 7.2 citations per document. Both journals are in Quartile Score 2.

In the final four positions are “AIP Conference Proceedings” with 21 publications, “Journal of Building Engineering” with 20 publications, “Advanced Materials Research” with 16 publications, and “Waste Management” with 16 publications. The “Journal of Building Engineering” and “Waste Management” both have a local H-Index of 15 and are in Quartile Score Q1, ranking second and fourth in terms of citations. However, “Waste Management” has the highest average citations per document among all the journals, indicating that its publications have a greater impact.

Consequently, the most significant impact of journals, such as Construction and Building Materials, Journal of Cleaner Production, Waste Management, and Journal of Building Engineering, demonstrates how closely the construction sector is linked to sustainability and waste management. As construction is one of the most polluting sectors, there is a clear concern and effort to reduce its associated environmental impacts.

**Figure 8:**
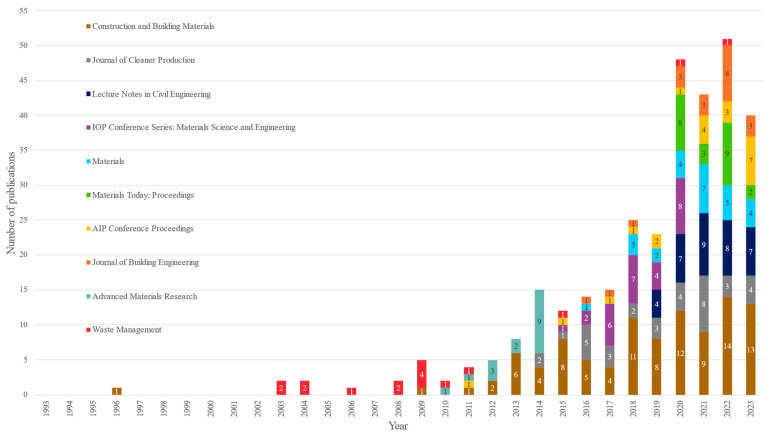
Evolution of the top 10 scientific journal publications over the past 30 years. The graph illustrates how certain journals have consistently published annually with an increase in output, while others have newly entered this field.

Figure 8 illustrates the temporal evolution of publications from key journals, revealing a notable increase in contributions since 2012 onwards. This trend suggests a link to the Sustainable Development Goals (SDGs) and underscores the role of regulatory frameworks in promoting waste management within the construction sector. While only four journals published on this topic between 1993 and 2014, the number has grown steadily since 2012, confirming an upward trajectory in research interest within this field.

### 3.7. Bibliometric Evolution in Research Areas

**Figure 9:**
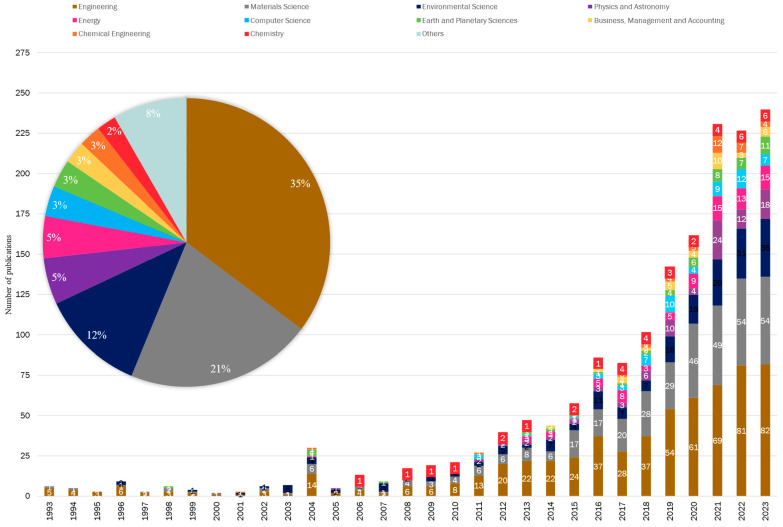
Distribution and temporal evolution of the ten research areas with the highest number of documents between 1993 and 2023, represented in the linear graph. In the bar chart, numbers below 3 are not shown for improved visibility.

The analysis of research areas was based on documents by subject area from the Scopus database. According to these categories, a total of 23 research areas converge in the study of WRTs in building and construction materials. The three most prominent areas (based on the number of publications) are engineering, material science and environmental science, with 36%, 21% and 12% of the total publications, respectively. Figure 9 depicts these research areas and their temporal evolution over the last 30 years. Apart from the three main areas, Energy and Physics and Astronomy each account for 5% of the publications, while Computer Science, Earth and Planetary Sciences, Business, Management and Accounting and Chemical Engineering each account for the 3%.

The aforementioned 2002 event, probably related to the European Union’s Landfill Directive, clearly stands out in Figure 9, with a sudden but temporary spike of research areas publishing in WRTs in 2004. The publishing delay between an event and its publication date, which could be argued is of the order of one year, is in agreement with the timing of this fluctuation.

From the data, it can be concluded that the use of recycled tyres in construction has gained significant momentum in recent years, underscoring the growing interest in key areas of study: engineering, material science, and environmental science. Additionally, a multidisciplinary approach is emerging, integrating various other fields of research.

### 3.8. Keyword Analysis

The analysis of keywords and their frequencies provides insights into the identification of research trends and the assessing of research impacts [25,91]. In order to evaluate the WRT-relevant keywords and their temporal evolution, the search has been limited to the last six years [2017, 2022], using “co-occurrence” and “author keywords” fields in VOSviewer software, setting a minimum of five occurrences. Out of the 2132 available keywords, 106 met the threshold. The last six years have been selected as the most representative in the evolution of keywords.

**Figure 10:**
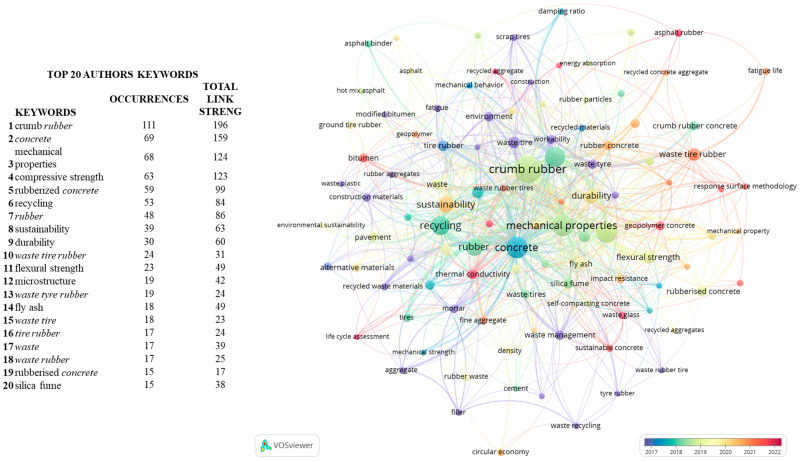
Evolution of keywords grouped by clusters, indicating the total number of occurrences and the links indicating the relationship they have with other keywords. The circle diameter represents the frequency of each word occurrence. In the list of keywords, the terms used in the bibliometric search are shown in italics. Made with VOSviewer software.

Figure 10 illustrates the relative frequency of these 106 keywords which were identified with a total of 1398 occurrences, among which the most frequent are “crumb rubber”, “concrete”, “mechanical properties”, “recycling”, and “compressive strength”.

At the beginning of the studied period, by 2017, the highest frequency keywords are “waste material”, “waste rubber”, and “waste recycling”, highlighting a focus on the reuse and recovery of waste. By 2018, the keywords shift towards mechanical properties such as “compressive strength”, “density”, “flexural strength”, and “alternative materials”, indicating a trend towards testing and mechanical resistance of construction materials.”

In 2019, we find keywords such as “fracture energy”, “rubbercrete”, “fatigue life”, “mechanical property”, “recycled concrete aggregate”, and “energy absorption”, reflecting a focus on testing the behaviour of crumb rubber from tyres (CRT) as a recycled aggregate in construction elements. In 2020, keywords like “sustainability” and “rubber concrete” highlight the emergence of new materials with environmental considerations. In 2021, terms such as “circular economy” and “waste tire rubber” emerge, emphasising a shift from a linear economic model to a circular one in relation to CRT. By 2022, keywords such as “life cycle assessment”, “thermal conductivity”, “sustainable concrete”, “geopolymer concrete”, and “sound absorption” reflect the sector’s evolution towards reducing environmental impacts.

**Figure 11:**
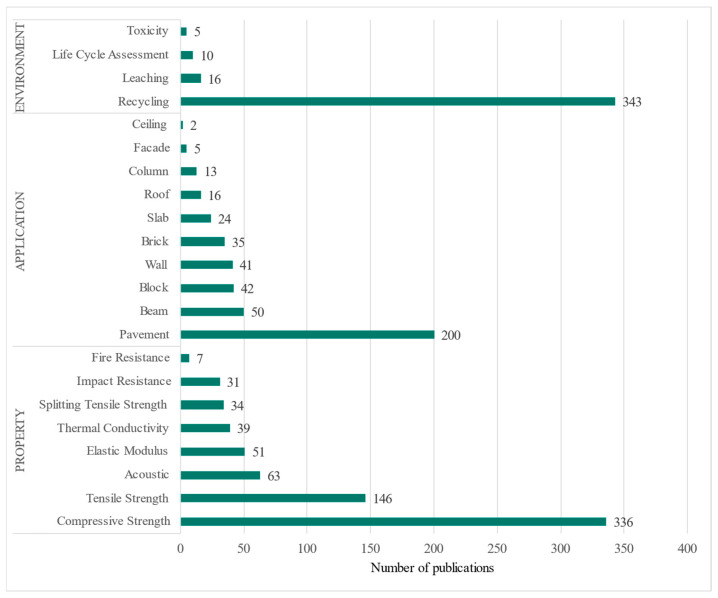
The chart shows the number of publications on topics related to the use of tyre waste in construction, organised into three main categories: environment, application, and property.

To conduct a more thorough analysis and identify gaps in the research, a targeted search was performed by adding the keywords shown in Figure 11 to the search query. The aim was to determine within the context of waste rubber tyres (WRTs) in building and construction materials whether research has focused on or considered the application of new materials, how many documents in our samples address environmental issues, or if there is merely a trend towards testing the material.

The results of the targeted search focusing on “environment”, “application”, and “property” reveal an interest in testing mechanical resistances, such as compression. However, since CRT is a highly flammable waste, only seven documents addressing this issue were found. Regarding “environmental statement”, recycling is the most discussed topic, but aspects like toxicity and leaching are less studied.

In accordance with the 2023 Synthetic Polymer Microparticle regulation, which restricts the release of microplastics into the environment, products made from tyres must consider these regulations when exploring potential applications. A particular case is the use of recycled rubber granules from tyres in artificial turf, the use of which has been restricted by the European Union due to the composition of tyre microfragments. When in contact with the soil, these fragments may leach, potentially causing contamination and toxicity in aquifers and the environment. Few studies have highlighted the risks associated with using this waste in applications such as artificial turf, indicating that leaching could pose a risk to human health through dermal exposure, oral ingestion, and inhalation [92,93]. Given that these artificial turfs have been in use for several years, it will be necessary to remove them and replace them with more sustainable alternatives in accordance with established regulations.

It is also worth noting that the regulation sets minimum sizes for the microparticles that can be used in the manufacture of materials, in order to prevent their release into the environment. Additionally, encapsulated microplastics are considered to pose no risk of release into the environment. This means that, when used, such as in the case of replacing aggregates, they do not cause negative effects. According to the studies reviewed, it has been found that, so far, no leaching has been observed in tyres encapsulated in asphalt and concrete [94,95]. However, this remains a relevant issue that must be considered when incorporating this waste into construction materials. Consequently, the need for further research in this area is recommended.

As observed in the research areas analysis, topics such as chemistry are beginning to emerge, suggesting a need for more research in these areas. Data from Figure 11 highlight some gaps and potential studies that can be carried out in the areas of the environment, material properties and new possible applications. The keyword analysis of the 926 documents found indicates that 36% discuss “compressive strength”, 37% focus on “recycling”, and 26% mention “pavement”.

Recycled rubber has found some interesting applications in construction, especially in pavements, where it is used in asphalt mixes and concrete to enhance flexibility and abrasion resistance [96]. Although its use in blocks and walls has been less prominent compared to pavements, promising results have been obtained: rubber improves acoustic insulation and impact absorption, while also making blocks and panels lighter than conventional ones [97]. Similarly, its incorporation in columns and beams has been limited, as rubber tends to reduce compressive strength, restricting its application in structural elements [98].

Research into its use in roofs and facades is also scarce, although some studies suggest potential for recycled rubber in non-structural applications [12,99,100]. It is important to note that various innovative applications for WRT are being explored by researchers. These include anti-collision concrete [101], aggregates for 3D printing mortar [102], permeable concrete [103], extruded cement [104], sandwich-type pavement aggregates [105], and acoustic insulation boards in slabs [106]. Overall, there are significant opportunities identified in the realm of applications.

Research on new materials incorporating waste should contribute to new construction applications and move beyond the academic sphere, seeking market opportunities. In this regard, trends towards use in pavements were detected, although this does not diminish the potential for using the waste in various other applications.

## 4. Conclusions

The present bibliometric study was chiefly based on data from Scopus, although it was previously cross-checked with Web of Science. Given the broader nature of the sample offered by Scopus, the analyses were carried out using this database, a decision which may generate some bias. However, given the status of Scopus as a standard reference in the academic community, its results are considered representative and relevant. It is recommended that future research endeavours extend the present scope by incorporating additional databases. This will facilitate the verification of the conclusions presented here and may also provide further insights that could lead to more comprehensive clarification.

The bibliometric analysis of waste rubber tyre (WRT) utilisation in the building and construction sector reveals several key trends and research areas in the scientific literature. An exponential increase in the number of publications is observed, showing a growth rate of 17% per year. Moreover, geographic data show that WRT research is unequally present on all continents. While India and China stand out with 13% and 10% of the publications, respectively, Africa and Central America fall behind, suggesting that the statistics of this topic are dominated by generalised Pareto dynamics, with most of the resources and benefits concentrated among a minority of agents.

One possible reason for the increase in tyre research in India and China is the implementation of Extended Producer Responsibility (EPR) regulations, which include end-of-life tyres (ELTs). In China specifically, the 2017 China Waste Import Ban and circular economy policies appear to have further stimulated research in the ELT field. Additionally, global ELT management initiatives promote the circular economy for tyres across various countries.

Moreover, India and China lead in the number of publications, whereas the USA leads in the impact ranks. The publishing output of a country positively correlates with its gross domestic product (GDP), but only moderately. A negative correlation of publication volume with the international co-collaboration percentage is obtained, mainly due to India’s contrast between its emphasis on education and its problems to acquire international projection. Surprisingly, Germany, Japan and France have a minimal presence in WRT research compared to the top 10 countries in publications, despite being leaders in car and tyre production.

According to this study, the correlation of the total number of publications with the total number of citations is positive and of moderate magnitude, while its correlation with the average citations per document is notably negative, leading to the conclusion that the impact of a country is not tied to a high volume of articles.

Correlations between the number of citations and the rest of the metrics are, in the majority of instances, lower when measured by rank. In particular, the Pearson vs. Kendall correlation coefficients between the total number of publications and the average number of citations are 0.73 and 0.30, respectively, revealing that a high rank in the number of citations does not necessarily lead to a high rank in the average citations per document.

Additionally, a high correlation between the global and the local impact of each publication indicates that local metrics are a good proxy for their global counterparts. And similarly, the ratio between local and global metrics reveals that WRT is a field with similar or better engagement metrics than other fields developed by the same top publishing authors.

While Cite score 2023, Scimago Journal and Country Rank (SJR) 2023 and Source Normalised Impact per Paper (SNIP) 2023 are strongly correlated, the number of published papers and their average citation numbers seem to be completely independent, leading to the conclusion that the publication volume of a journal does not correlate with its impact.

Not only has the use of recycled tyres in construction gained momentum in recent years, highlighting the interest in the main areas of study (engineering, material science, and environmental science), but a multidisciplinary approach is also emerging, involving other fields of research.

Among the most cited publications, the focus is primarily on the mechanical properties of the material, with limited attention given to its practical applications in construction. While significant efforts have been made to develop the material, there is a clear gap in research exploring its use in construction elements. This underscores the need for further studies that not only enhance the material’s properties but also investigate its potential applications within the industry.

The keyword analysis has shown a clear evolution in the handling of WRTs, moving from initial recycling efforts to its subsequent integration into construction. Among the identified themes, there are gaps and opportunities related to environmental issues, particularly in research concerning toxicity, leaching, and life cycle analysis.

Regarding the properties of materials developed with ELTs, there is a large number of studies focused on compressive strength. However, there remains a significant gap in studies addressing fire resistance, impact resistance, and thermal conductivity of the material, presenting a valuable opportunity for future research.

In terms of the application of ELT in construction elements, there is a substantial amount of research dedicated to its use in asphalt and concrete pavements. However, future research should explore the use of crumb rubber from tyres (CRT) as a partial or total substitute for natural fine aggregate (NFA) or natural coarse aggregate (NCA) in non-structural elements, such as facade elements, blocks, panels, walls, roofs, and more. The integration of rubber waste into construction materials holds great promise, not only in pavement applications but also in the creation of new products and building elements; therefore, it is important to continue exploring and developing innovative construction elements from waste.

In the initial searches, without including terms such as “building”, “construction”, or “concrete” in the keywords, it was observed that over 50% of publications on recycled tyres are related to engineering topics. This demonstrates a clear motivation to incorporate these wastes into the construction sector. Despite being one of the major environmental offenders, due to the high demand for natural aggregates and its contribution to global emissions, the construction industry is making efforts to reduce environmental impacts by substituting raw materials with waste, aiming to decrease the embedded energy and emissions.

Nevertheless, the construction industry continues to generate large quantities of construction and demolition waste (CDW), which, due to its heterogeneity, hinders the achievement of uniform properties in materials, especially in terms of strength. This limits its reuse as a substitute for raw materials, leaving a significant portion of these materials inadequately managed. Although there are global initiatives to reduce waste and promote cross-sector collaboration, it is crucial that each industry takes responsibility for managing its own waste, as the industry generating this waste has the appropriate knowledge to handle it efficiently. Therefore, each sector should focus on optimising the management of its own waste before assuming external responsibilities.

## Figures and Tables

**Table 1:** Results from the number of articles obtained from each database and the exact query used. The query was searched on 3 July 2024.

Databases	Search Results	Query
SCOPUS	926	TITLE-ABS-KEY ((BUILDING OR CONSTRUCTION OR CONCRETE) AND MATERIAL AND WASTE AND RUBBER AND (TIRE OR TYRE)) AND PUBYEAR > 1992 AND PUBYEAR < 2024 AND (LIMIT-TO (PUBSTAGE, “FINAL”)) AND (EXCLUDE (DOCTYPE, “NO”) OR EXCLUDE (DOCTYPE, “SH”) OR EXCLUDE (DOCTYPE, “TB”))
Web of Science Core Collection	863	REFINE RESULTS FOR (BUILDING OR CONSTRUCTION OR CONCRETE) AND MATERIAL AND WASTE AND RUBBER AND (TIRE OR TYRE) (TOPIC) AND 2024 (EXCLUDE–FINAL PUBLICATION YEAR) https://www.webofscience.com/wos/woscc/summary/e3dc33b2-a58b-4933-83cd-4b0b636e9aee-f87b8a15/relevance/1 (accessed on 3 July 2024)

**Table 2:** Top ten countries by number of citations.

Country	Total Publications	Total Cited	Average Citations per Document	Local H-Index	International Co-Authorship (%)
India	144	2896	20.1	24	16
China	112	2664	23.8	28	40
USA	79	5395	68.3	29	45
Malaysia	57	1158	20.3	17	72
Australia	53	2844	53.7	20	47
Brazil	48	398	8.3	11	17
Italy	43	1615	37.6	19	37
UK	40	2290	57.3	18	68
Spain	39	1977	50.7	19	51
Iran	35	1788	51.1	17	57

**Table 3:** Pearson and Kendall’s correlation and rank-correlation coefficients for the metrics shown in Table 2. These symmetric matrices measure the degree of correlation between each pair of metrics. While Pearson’s coefficient takes the magnitude of the metrics into account, Kendall’s focuses on their ranks.

Pearson | Kendall	Total Publications	Number of Citations	Average Citations per Document	Local H-Index	International Co-Authorship (%)
Total publications	1.00 | 1.00	0.42 | 0.33	−0.37 | −0.24	0.67 | 0.43	−0.52 | −0.38
Number of citations	0.42 | 0.33	1.00 | 1.00	0.63 | 0.42	0.86 | 0.75	0.01 | −0.16
Average citations per document	−0.37 | −0.24	0.63 | 0.42	1.00 | 1.00	0.32 | 0.25	0.49 | 0.33
Local H-index	0.67 | 0.43	0.86 | 0.75	0.32 | 0.25	1.00 | 1.00	−0.07 | −0.30
International co-authorship	−0.52 | −0.38	0.01 | −0.16	0.49 | 0.33	−0.07 | −0.30	1.00 | 1.00

**Table 4:** Top 10 publishing affiliations.

Affiliation	Total Publications	Number of Citations	H-Index Local	Average Citations per Document	Country
Malaviya National Institute of Technology Jaipur (MNIT)	18	1410	10	78.3	India
Royal Melbourne Institute of Technology (RMIT University)	17	1068	8	62.8	Australia
Universiti Tun Hussein Onn Malaysia (UTHM)	15	165	8	11.0	Malaysia
Southeast University (SEU)	14	204	6	14.6	China
Universiti Teknologi Malaysia (UTM)	13	357	5	27.5	Malaysia
Islamic Azad University	12	421	7	35.1	Iran
Sapienza University of Rome	12	329	6	27.4	Italy
Universidade Estadual de Campinas (UNICAMP)	12	101	2	8.4	Brazil
Czech Technical University in Prague (CTU)	11	118	3	10.7	Czech Republic
Ministry of Education of The People’s Republic of China	11	324	4	29.5	China

**Table 5:** Top 10 most published authors.

Author	Local				Global/Scopus Metric			Affiliations
	Number of Publications	Total Number of Citations	Average Citations per Document	Local H-Index	Global H-Index	Number of Publications	Total Number of Citations	
Marco Valente	12	329	27.4	11	21	68	1843	Sapienza Università di Roma, Rome, Italy
Sandeep Chaudhary	11	490	44.5	8	29	120	3489	Indian Institute of Technology Indore, Indore, India
Rosa C. Cecche Lintz	11	50	4.5	4	9	52	517	Universidade Estadual de Campinas, Campinas, Brazil
Matteo Sambucci	11	296	26.9	10	14	31	491	Sapienza Università di Roma, Rome, Italy
Jie Li	10	303	30.3	8	41	204	4432	RMIT University, Melbourne, Australia
Zbyšek Pavlík	9	118	13.1	4	35	363	3803	Czech Technical University in Prague, Prague, Czech Republic
Milena Pavlíková	9	118	13.1	4	28	267	2494	Czech Technical University in Prague, Prague, Czech Republic
Mohammad Saberian	8	219	27.4	6	37	95	2923	RMIT University, Melbourne, Australia
Shahiron Shahidan	8	95	11.9	6	25	210	2186	Universiti Tun Hussein Onn Malaysia, Batu Pahat, Malaysia
Yan Zhuge	7	403	57.6	5	40	253	5989	University of South Australia, Adelaide, Australia

**Table 6:** Top 10 most globally cited documents.

Year	Document	Author	DOI	Local Citations	Global Citations	Cited Ref
2004	Properties of concrete containing scrap-tire rubber: an overview	Siddique, Rafat and Naik, Tarun R	10.1016/j.wasman.2004.01.006	121	793	[82]
1999	Rubberised Portland Cement Concrete	Zaher K. Khatib and Fouad M. Bayomy	10.1061/(ASCE)0899-1561(1999)11:3(206)	106	665	[83]
2008	Mechanical properties of concrete containing a high volume of tire-rubber particles	Khaloo, Ali R and Dehestani, M and Rahmatabadi, P	10.1016/j.wasman.2008.01.015	92	603	[84]
2010	Waste tyre rubberised concrete: Properties in fresh and hardened state	Aiello, M. A., & Leuzzi, F.	10.1016/j.wasman.2010.02.005	59	296	[85]
2014	Hardened properties of concrete mixtures containing pre-coated crumb rubber and silica fume	Onuaguluchi, O., & Panesar, D. K.	10.1016/j.jclepro.2014.06.068	58	298	[86]
2013	Utilisation of recycled crumb rubber as fine aggregates in concrete mix design	Issa, C. A., & Salem, G. (2013).	10.1016/j.conbuildmat.2012.12.054	49	216	[87]
1996	Cement-based materials containing shredded scrap truck tyre rubber	Fattuhi, N. I., and L. A. Clark.	10.1016/0950-0618(96)00004-9	48	316	[88]
2008	Mechanical, fracture, and microstructural investigations of rubber concrete	Reda Taha, Mahmoud M., et al.	10.1061/(ASCE)0899-1561(2008)20:10(640)	46	396	[89]
2014	Strength, abrasion and permeation characteristics of cement concrete containing discarded rubber fine aggregates.	Thomas, B. S., Gupta, R. C., Kalla, P., & Cseteneyi, L.	10.1016/j.conbuildmat.2014.01.074	45	249	[70]
2008	Promoting the use of crumb rubber concrete in developing countries.	Batayneh, M. K., Marie, I., & Asi, I.	10.1016/j.wasman.2007.09.035	43	313	[90]

**Table 7:** Top 10 journal productions/conference publications.

Journal/Conferences	Total Local				Journal Quality				
	Paper	Citations	Average Citations per Document	H-Index	H-Index	Cite Score 2023	SJR 2023	SNIP 2023	Quartile Score 2023
				Local	Global				
Construction and Building Materials	100	6449	64.5	47	259	13.8	1.999	2.11	Q1
Journal of Cleaner Production	35	2738	78.2	28	309	20.4	2.058	2.236	Q1
Lecture Notes in Civil Engineering **	35	55	1.6	5	25	0.8	0.162	0.243	Q4
IOP Conference Series: Materials Science and Engineering *	28	247	8.8	9	62	1.1 (2021)	0.249 (2021)	0.605	-
Materials	26	612	23.5	13	168	5.8	0.565	0.979	Q2
Materials Today: Proceedings	22	158	7.2	8	88	4.9	0.473	0.805	Q2
AIP Conference Proceedings *	21	35	1.7	3	83	0.5	0.152	0.291	-
Journal of Building Engineering	20	962	48.1	15	92	10	1.397	1.936	Q1
Advanced Materials Research **	16	46	2.9	4	52	-	0.121	0.182	Q4 (2013)
Waste Management	16	3157	197.3	15	220	15.6	1.734	1.804	Q1

* Journals whose publications consist of conference papers and conference reviews, thus the document only provides abstracts. ** Journals whose publications include conference papers, reviews and book chapters.

**Table 8:** Shows the Pearson’s and Kendall’s coefficient matrices for these metrics.

Pearson | Kendall	Number of Papers	Number of Citations	Average Citations per Document	Local H-Index	Global H-Index	Cite Score 2023	SJR 2023	SNIP 2023
Number of papers	1.00 | 1.00	0.81 | 0.30	0.05 | 0.02	0.86 | 0.28	0.47 | 0.11	0.33 | 0.16	0.47 | 0.20	0.42 | 0.20
Number of citations	0.81 | 0.30	1.00 | 1.00	0.60 | 0.82	0.94 | 0.94	0.80 | 0.64	0.75 | 0.78	0.85 | 0.82	0.77 | 0.73
Average citations per document	0.05 | 0.02	0.60 | 0.82	1.00 | 1.00	0.40 | 0.76	0.66 | 0.73	0.77 | 0.78	0.75 | 0.73	0.69 | 0.73
Local H-index	0.86 | 0.28	0.94 | 0.94	0.40 | 0.76	1.00 | 1.00	0.81 | 0.67	0.75 | 0.81	0.84 | 0.85	0.81 | 0.81
Global H-index	0.47 | 0.11	0.80 | 0.64	0.66 | 0.73	0.81 | 0.67	1.00 | 1.00	0.92 | 0.78	0.89 | 0.82	0.84 | 0.82
Cite Score 2023	0.33 | 0.16	0.75 | 0.78	0.77 | 0.78	0.75 | 0.81	0.92 | 0.78	1.00 | 1.00	0.97 | 0.96	0.95 | 0.87
SJR 2023	0.47 | 0.20	0.85 | 0.82	0.75 | 0.73	0.84 | 0.85	0.89 | 0.82	0.97 | 0.96	1.00 | 1.00	0.98 | 0.91
SNIP 2023	0.42 | 0.20	0.77 | 0.73	0.69 | 0.73	0.81 | 0.81	0.84 | 0.82	0.95 | 0.87	0.98 | 0.91	1.00 | 1.00

## Supplementary Materials

The following supporting information can be downloaded at: https://www.mdpi.com/article/10.3390/polym17182480/s1, Table S1: PRISMA 2020 for Abstracts Checklist. The complete bibliometric and bibliography used in this study are openly available in https://futur.upc.edu/40242359, accessed on 11 December 2024.
